# Spatial and single-cell explorations uncover prognostic significance and immunological functions of mitochondrial calcium uniporter in breast cancer

**DOI:** 10.1186/s12935-024-03327-z

**Published:** 2024-04-17

**Authors:** Chia-Jung Li, Yen-Dun Tony Tzeng, Jui-Hu Hsiao, Ling-Ming Tseng, Tzu-Sheng Hsu, Pei-Yi Chu

**Affiliations:** 1 Department of Obstetrics and Gynecology, Kaohsiung Veterans General Hospital, Kaohsiung, 813 Taiwan; 2 Institute of BioPharmaceutical Sciences, National Sun Yat-sen University, Kaohsiung, 804 Taiwan; 3 Department of Surgery, Kaohsiung Veterans General Hospital, Kaohsiung, 813 Taiwan; 4 Institute of Biomedical Sciences, National Sun Yat-sen University, Kaohsiung, 804 Taiwan; 5 Department of Surgery, Kaohsiung Municipal Minsheng Hospital, Kaohsiung, 802 Taiwan; 6 School of Medicine, National Yang-Ming University, Taipei, 112 Taiwan; 7 Comprehensive Breast Health Center, Taipei Veterans General Hospital, Taipei, 112 Taiwan; 8 Institute of Molecular and Cellular Biology, College of Life Sciences and Medicine, National Tsing Hua University, Hsinchu, 300 Taiwan; 9 Department of Post-Baccalaureate Medicine, College of Medicine, National Chung Hsing University, Taichung, 402 Taiwan; 10 School of Medicine, College of Medicine, Fu Jen Catholic University, New Taipei City, 242 Taiwan; 11 Department of Pathology, Show Chwan Memorial Hospital, Changhua, 500 Taiwan; 12 National Institute of Cancer Research, National Health Research Institutes, Tainan, 704 Taiwan

**Keywords:** MCU, Immune infiltration, Breast cancer, Single-cell RNA-sequencing, Spatial transcriptomics

## Abstract

The mitochondrial calcium uniporter (MCU) is a transmembrane protein facilitating the entry of calcium ions into mitochondria from the cell cytosol. Maintaining calcium balance is crucial for enhancing cellular energy supply and regulating cell death. The interplay of calcium balance through MCU and the sodium-calcium exchanger is known, but its regulation in the breast cancer tumor microenvironment remains elusive. Further investigations are warranted to explore MCU’s potential in BRCA clinical pathology, tumor immune microenvironment, and precision oncology. Our study, employing a multi-omics approach, identifies MCU as an independent diagnostic biomarker for breast cancer (BRCA), correlated with advanced clinical status and poor overall survival. Utilizing public datasets from GEO and TCGA, we discern differentially expressed genes in BRCA and examine their associations with immune gene expression, overall survival, tumor stage, gene mutation status, and infiltrating immune cells. Spatial transcriptomics is employed to investigate MCU gene expression in various regions of BRCA, while spatial transcriptomics and single-cell RNA-sequencing methods explore the correlation between MCUs and immune cells. Our findings are validated through the analysis of 59 BRCA patient samples, utilizing immunohistochemistry and bioinformatics to examine the relationship between MCU expression, clinicopathological features, and prognosis. The study uncovers the expression of key gene regulators in BRCA associated with genetic variations, deletions, and the tumor microenvironment. Mutations in these regulators positively correlate with different immune cells in six immune datasets, playing a pivotal role in immune cell infiltration in BRCA. Notably, high MCU performance is linked to CD8 + T cells infiltration in BRCA. Furthermore, pharmacogenomic analysis of BRCA cell lines indicates that MCU inactivation is associated with increased sensitivity to specific small molecule drugs. Our findings suggest that MCU alterations may be linked to BRCA progression, unveiling new diagnostic and prognostic implications for MCU in BRCA. The study underscores MCU's role in the tumor immune microenvironment and cell cycle progression, positioning it as a potential tool for BRCA precision medicine and drug screening.

**Supplementary Information:**

The online version contains supplementary material available at 10.1186/s12935-024-03327-z.

## Introduction

Breast cancer (BRCA) has been the most prevalent malignancy among women globally. Recent studies have highlighted a rise in the incidence of breast cancer among younger individuals [1]. Despite advancements, drug resistance, recurrence, and metastasis have remained primary contributors to treatment failure [2, 3]. Clinical categorization of breast cancer into Luminal A, Luminal B, HER2 overexpression, and triple-negative subtypes, based on ER, PR, HER2, and Ki67, has guided therapeutic decisions [4, 5]. However, breast cancer's complexity and heterogeneity across individuals have posed significant challenges [6, 7]. Currently, surgery, radiation, and endocrine therapy constitute the mainstay treatments for BRCA. Neoadjuvant therapy, which combines chemotherapy with targeted drugs, is widely used for high-risk BRCA patients [8]. Unfortunately, resistance commonly develops, leading to inevitable relapse. Hence, there is a critical need for reliable biomarkers that facilitate accurate diagnosis, prognosis, and therapeutic targeting in BRCA.

Serving as a pivotal organelle within cells, mitochondria possess an intricate mechanism to maintain Ca^2+^ homeostasis. Besides the voltage-dependent Ca^2+^ channels present in the outer mitochondrial membrane, the MCU stands out as the principal pathway responsible for the uptake of Ca^2+^ into mitochondria [9]. The MCU not only governs mitochondrial Ca^2+^ levels but also exerts regulatory control over intracellular signaling cascades, which converge at this organelle, ultimately influencing cellular processes such as proliferation, differentiation, and apoptosis. The MCU complex, which plays a crucial role in mitochondrial calcium uptake, consists of several key components. These include the pore-forming subunit, MCU, responsible for facilitating calcium ion entry into the mitochondria. The complex also includes MCUb, an inhibitory form of MCU, adding a regulatory layer to the calcium transport process [10]. Furthermore, the essential MCU regulator (EMRE) is a vital component that modulates and regulates the activity of the MCU complex [11]. Additionally, the regulatory subunits for mitochondrial calcium uptake, MICU1, MICU2, and MICU3, contribute to fine-tuning the transport of calcium ions into the mitochondria, ensuring precise control of intracellular calcium homeostasis [12].

The expression of Mitochondrial Calcium Uniporter (MCU) and p38 correlated positively with glioma grading and tumor progression. MCU enhanced the migration of glioma cells by upregulating p38. Additionally, p38 promoted glioma progression through autophagy mediated by transcription factor EB (TFEB) [13]. Furthermore, calcium concentration-mediated regulation of mitochondrial fatty acid metabolism had a specific impact on MDA-MB-231 cell migration [14]. High levels of MCU expression were also observed in ovarian cancer, and its silencing reduced proliferation and migration of ovarian cancer cells, possibly due to reduced ROS production [15]. In colorectal cancer, MCU was targeted by miR-138-5p, with downregulation of miR-138-5p leading to increased MCU expression [16]. Moreover, elevated mitochondrial calcium uptake was associated with metastasis formation in pancreatic ductal adenocarcinoma (PDAC). The role of MCU in PDAC metastasis involved activation of the KEap-Nrf2 antioxidant pathway. This study suggested that MCU may have also played a role in the pathogenesis of pancreatic cancer, particularly through its structural subunit EMRE [17].

Notably, mitochondria play a crucial role in orchestrating T cell activities. Upon T cell activation, mitochondria undergo remodeling and polarization near the immune synapse, ensuring an adequate supply of ATP to meet the heightened demands of activated cells [18]. Additionally, mitochondria function as buffers for Ca^2+^, preventing Ca^2+^-dependent channel inactivation and maintaining a sustained Ca^2+^ influx critical for T cell functions [19]. This dynamic interplay highlights the central role of mitochondria in shaping the activation, metabolism, and functional outcomes of T cells in the immune response.

This study conducted a comprehensive analysis to investigate the diagnostic potential of MCU in breast cancer (BRCA). Initial assessments included differential gene expression, diagnostic efficacy, and prognostic value of MCU. Furthermore, we explored the association between MCU manifestations and the tumor microenvironment (TIME) at both single-cell and whole-tissue levels. Our study elucidated the potential mechanism behind MCU's tumor-promoting effects through functional enrichment modules, with experimental validation. The clinicopathological significance of MCU was confirmed using real-world BRCA patient tissues and pan-cancer bioinformatics. Additionally, by leveraging data from the Genomics of Drug Sensitivity in Cancer (GDSC) and Cancer Cell Line Encyclopedia (CCLE) repositories, MCU emerged as a validated drug candidate for targeting cancer cells with elevated MCU expression.

## Materials and methods

### Comprehensive multi-omics exploration of differential gene expression and prognostic significance

To delve into the landscape of gene mutations, DNA copy number alterations (CNAs), gene domain variations, and mRNA expression, a thorough bioinformatics analysis was conducted, employing well-established methodologies [20, 21]. Data from diverse platforms, including TNMplot [22], UALCAN Portal [23], and Gene Expression Omnibus (GEO), were harnessed to assess gene expression levels in breast cancer (BC) tumors and adjacent normal tissues. Gene Expression Profile Interaction Analysis 2 (GEPIA2) [24, 25], utilizing TCGA and Genotype-Tissue Expression (GTEx) data, was instrumental in identifying Differentially Expressed Genes (DEGs), employing a threshold of absolute fold change (FC) > 1. DEGs of particular interest underwent further scrutiny. For survival analysis, the Kaplan–Meier plotting tool was employed to scrutinize the correlation between clinical stage in breast cancer (BRCA) and various factors, including immune cell content and tumor mutational burden. The tool was configured to autonomously determine the optimal cutoff for patient grouping in the survival analysis [26]. This comprehensive approach aimed to provide a nuanced understanding of the molecular landscape and prognostic implications in breast cancer.

### Human breast cancer specimens

This study follows previous publications, which are briefly described below. The specimens and related clinical data we used were obtained from the biobank database of Kaohsiung Veterans General Hospital [27]. This database includes essential patient history details and information on cancer stage. Ethical approval for all clinical studies was obtained in compliance with the regulations of the Biobank Ethics Governance Council of Kaohsiung Veterans General Hospital (Approval Code: KSVGH22-003).

### Integration of single-cell RNA-sequencing data processing and immunological profiling

The utilization of single-cell RNA-sequencing (scRNA-seq) has become a powerful method for exploring cellular diversity and gene expression at the individual cell level. In our study, scRNA-seq data from the GEO database underwent rigorous quality control (QC) using the R package Seurat. This ensured the inclusion of high-quality cells while minimizing batch-related variations. Employing uniform manifold approximation and projection (UMAP) clustering with the “BiocManager” and Gene Set Variation Analysis (“GSVA”) packages in R, we identified unique cellular subgroups. The annotation of these cell types involved comparing expression patterns with well-established cellular marker genes, using the “SingleR” package in R. Additionally, following a previously established methodology, we utilized TIMER datasets to examine potential relationships between the expression of various MCU genes and immune cell infiltration levels in BRCA. We also explored the correlation between MCU expression and genetic markers associated with tumor-infiltrating immune cells. This comprehensive analysis integrated single-cell transcriptomics with immune profiling, providing valuable insights into the intricate landscape of cellular heterogeneity and immune responses in breast cancer. Furthermore, employing a previously validated approach, we employed datasets from the Tumor Immune Estimation Resource (TIMER) to explore potential associations between the expression of diverse MCU genes and the levels of immune cell infiltration in BRCA. TIMER furnishes a comprehensive profile of various infiltrating immune cells, including B cells, CD4 + T cells, CD8 + T cells, neutrophils, macrophages, and dendritic cells, within tumor tissues, as identified by RNA-Seq expression profiling data. Adhering to a well-established methodology, we assessed potential connections between the expression of various MCU genes and immune cell infiltration levels in BRCA using TIMER datasets. Additionally, we delved into the correlation between MCU expression and genetic markers linked to tumor-infiltrating immune cells. This all-encompassing analysis seamlessly integrated single-cell transcriptomics with immune profiling, offering valuable insights into the complex landscape of cellular heterogeneity and immune responses in BRCA.

### Processing of spatial transcriptome data

In this investigation, we conducted an analysis of spatial transcriptomic data previously acquired from a study (STDS0000049). We summarized unique molecular identifiers (UMIs) in each bin100-defined point and performed cluster annotation based on hematoxylin and eosin (H&E) slices. Subsequently, additional annotation was carried out using cell markers. The resulting data were analyzed and visually represented to ascertain the expression levels and spatial distribution of MCU, AHR, FOXP3, ID2, IL10, IL21, IRF4, TGFb1, and STAT4. Clustering and dimensionality reduction of similar spatial transcriptomes were achieved using the RunPCA, FindNeighbors, and FindClusters functions. Given that certain clusters exhibited elevated expression of multiple cell markers, we employed the single-sample gene set enrichment analysis algorithm to score common cell types based on the average expression matrix of distinct clusters. This methodology has proven to be more effective in the context of spatial transcriptomes [28].

### Batch effect removal

Batch effects are a common challenge in datasets containing multiple donors or samples, leading to notable variability among the samples. To assess these effects, we utilized a metric that considers both information entropy and Euclidean distance within the UMAP graph for each dataset. Higher information entropy values indicate a more even distribution of batches. The formula for calculating entropy is provided.

$$Entropy\, = \, - \sum\limits_{1}^{N} {p_{n} } \log_{2} p_{n}$$

Upon closer examination of batch effects, we found that most datasets were impacted, resulting in significant variation across samples. To address this issue, we employed a metric based on information entropy and Euclidean distance within the UMAP graph. A higher entropy value suggests a better balance in batch mixing. If the ratio of the maximum entropy to the median entropy exceeded 4, we applied conventional correlation analysis (CCA) using Seurat v4.0.4 to mitigate batch effects.

### Human tissue microarrays and immunohistochemistry analysis

The research involved the analysis of human specimens through the utilization of tissue microarray (TMA) slides procured from SuperBioChips Laboratories in Seoul, Republic of Korea. These TMA slides encompassed samples from human breast cancer, metastatic cancer, and normal tissues. The immunohistochemistry (IHC) on the TMA slides was conducted following the established protocol outlined in a prior study [5, 29]. The IHC protocol entailed the use of specific antibodies to label and detect the proteins of interest within the tissue samples. Initial steps included fixation of samples with paraformaldehyde (4%) and permeabilization with Triton X-100 (0.2%). Subsequently, non-specific antibodies were blocked using BSA (5%) before the application of specific antibodies. Following an overnight incubation with primary antibodies at 4 °C, secondary antibodies were applied and left to incubate for 30 min at room temperature. The slides underwent examination and analysis at Li-Tzung Pathology Laboratory in Kaohsiung, Taiwan, with whole slide images captured using a BX61VS^®^ microscope manufactured by Olympus in Tokyo, Japan.

### Breast cancer gene-expression miner

Assess the expression and prognostic significance of ABCA10 in breast cancer through the Breast Cancer Gene-Expression Miner online dataset [30]. This online resource serves as a statistical mining tool for annotated transcriptomic data related to breast cancer, encompassing DNA microarray, RNA-seq, and RNA-seq datasets. The extensive collection of published annotated genomic data, particularly from RNA-seq, facilitates statistical analyses involving gene expression, correlation, and prognosis.

### *Anticipating drug response through MCU performance assessment *via* pharmacogenetics*

To examine drug sensitivity in relation to MCU expression, we employed two distinct datasets. Drug sensitivity profiling was conducted using an shRNA screening algorithm sourced from the Genomics of Drug Sensitivity in Cancer (GDSC) data repository. Additionally, we constructed a pharmacogenetic prediction model utilizing the CRISPR screening data repository of the GDSC algorithm. This model integrates publicly available data encompassing mutations, gene expression, patient survival, immune scores, drug screening, and RNAi screening, drawing from the CCLE, GDSC, and DepMap databases [5, 31].

### Statistical analysis

The total count of genes is largely influenced by the most prevalent genes, and their variability could significantly affect the scaling factor. To mitigate the impact of high-abundance genes on variability, alternative normalization methods such as median and upper quartile normalization have been suggested. These approaches estimate scaling factors using the 50 and 75 percentiles of the gene count distribution, respectively. This study employed established statistical methods [32]. Pearson's correlation coefficient assessed gene expression correlation, t-tests or Fisher's exact tests compared intergroup differences, and intragroup comparisons used one-way ANOVA. GraphPad Prism 8.0 software conducted statistical analysis, and significance was defined as p < 0.05.

## Results

### In-depth analysis unveils mutations within MCU genes in breast cancer

**Fig. 1:**
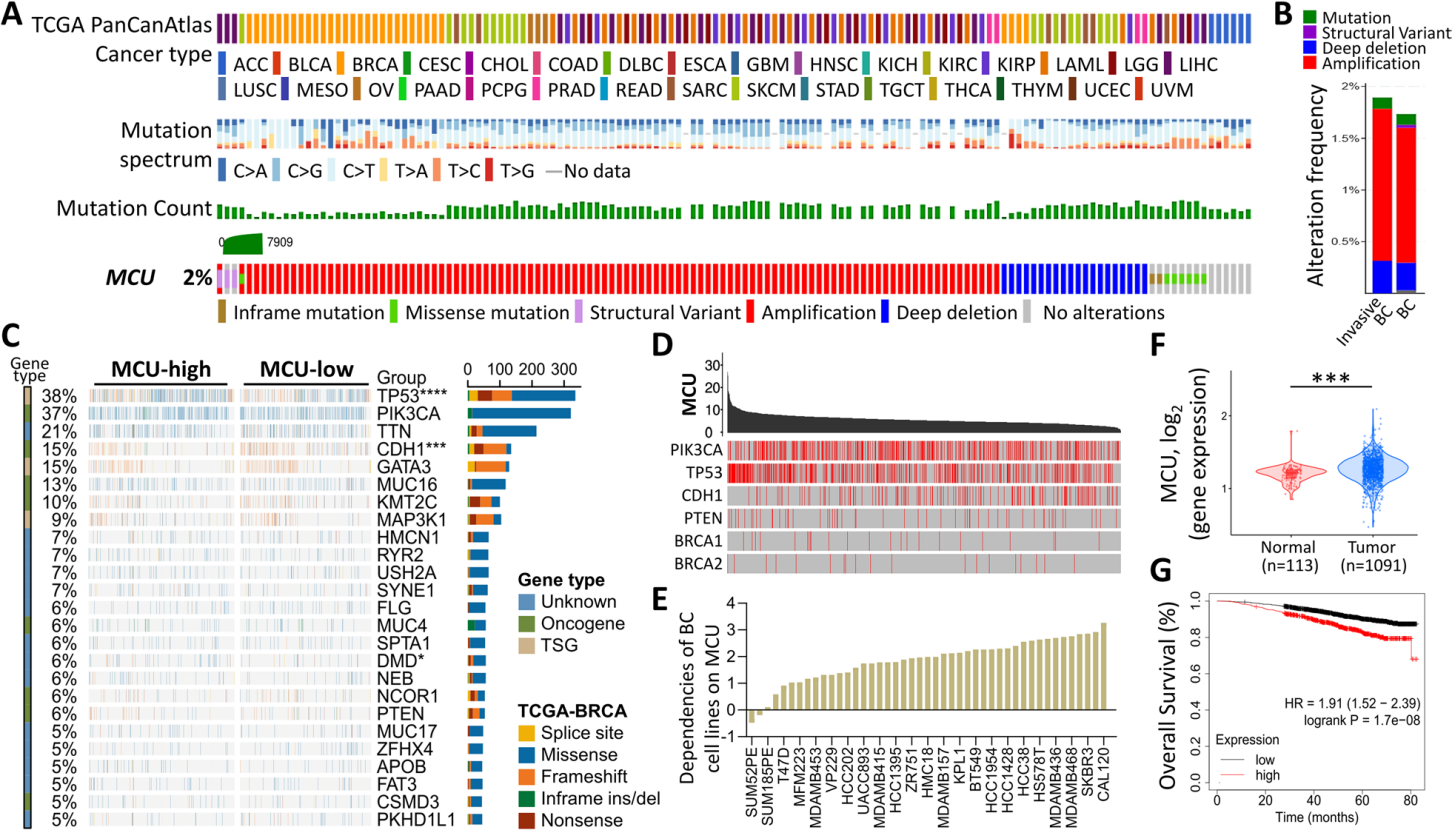
The illustrates the frequency and functional enrichment analysis of MCU alterations in breast cancer. **A** Examination of diverse mutations in the MCU gene across various cancer types. **B** Utilizing cBioPortal cancer genomics analysis to determine the frequency of MCU gene alterations in different cancer types. **C** Fisher's exact test compared mutation frequencies between MCU-high and MCU-low groups, with the right panel displaying mutation types, driving mutation types, and respective groups. **D** Investigation into the relationship between MCU and the six highly mutated genes in BRCA, with mutation sites highlighted by red lines. **E** Significance of MCU dependency in 45 BRCA cell lines based on the CRISPR screen. **F** Violin plots depicting MCU gene expression from RNA-sequencing data. **G** Kaplan–Meier survival analysis illustrating overall survival (OS) based on low/high expression of MCU. ***p < 0.001

Our initial exploration sought to ascertain the frequency and nature of MCU mutations in BRCA, leveraging TCGA’s BRCA dataset. The cBioPortal dataset revealed that MCU genes exhibit mutations in 2% of cancer cases, as illustrated in Fig. 1A. Subsequently, we delved into the amplification patterns of highly invasive breast cancers compared to general breast cancers within the TCGA dataset (Fig. 1B). Expanding our investigation, we extracted data on MCU expression in BRCA patients from the TCGA database and depicted it using a waterfall plot, specifically highlighting the top 25 affected genes (Fig. 1C). To deepen our understanding of MCU mutations in BRCA, we scrutinized their associations with other commonly observed cancer progression genes such as PIK3CA, TP53, and CDH1 (Fig. 1D). A comprehensive assessment of the dependence of 45 breast cancer cell lines on MCU ensued, with the MCU dependence of these cell lines presented in a ranked fashion based on increased MCU dependence (Fig. 1E). Further examination involved evaluating MCU expression across various clinical stages, revealing higher MCU expression in tumor tissues compared to non-tumor tissues (Fig. 1F). The impact of MCU on overall survival and disease-free survival in BRCA patients was analyzed using Kaplan–Meier plots, indicating an association between high MCU performance and poor prognosis (Fig. 1G).

### Assessing the link between MCU expression and clinicopathological parameters in BRCA

**Fig. 2:**
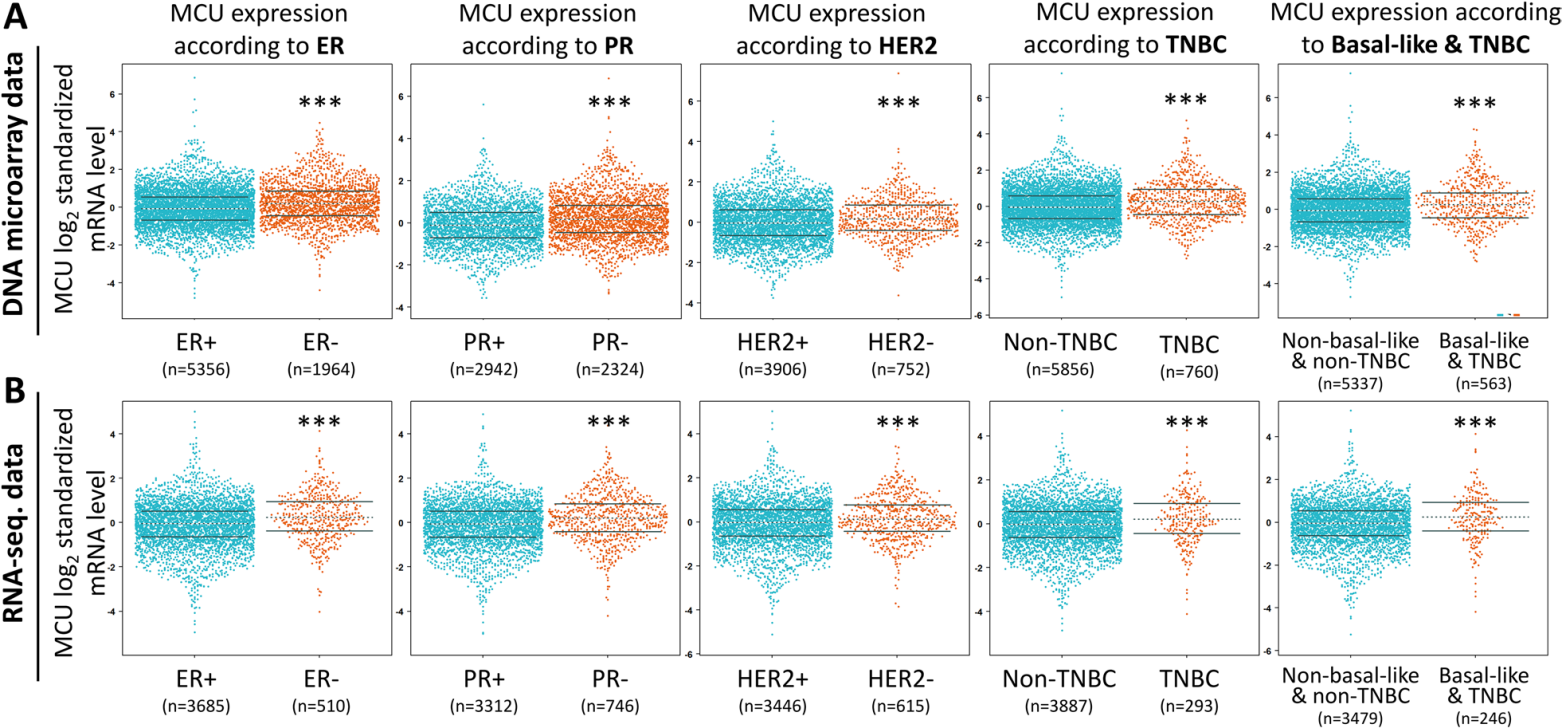
Bee swarm representation of differential expression in breast cancer patients based on various classified parameters. **A**–**B** Illustrate MCU mRNA expression levels in breast cancer patients using bee swarm plots in DNA microarray datasets and RNA-sequencing datasets. (*ER* estrogen receptor, *PR* progesterone receptor, *HER2* human epidermal growth factor receptor 2, *TNBC* triple-negative breast cancer)

To investigate the correlation between MCU performance and clinicopathological parameters in Breast Cancer (BRCA), we conducted analyses utilizing the bc-GenExMiner dataset. Both DNA microarray (Fig. 2A) and RNA sequencing data (Fig. 2B) consistently affirmed the elevated representation of MCU mRNA in Estrogen Receptor-positive (ER +) cases (ER− > ER + , p < 0.0001). Moreover, in the DNA microarray database, MCU mRNA exhibited a significantly higher representation in the Progesterone Receptor-negative (PR−) group compared to the Progesterone Receptor-positive (PR +) group (PR −  > PR + , p < 0.001). In the same database, MCU mRNA was notably upregulated in the Human Epidermal Growth Factor Receptor 2-positive (HER2 +) group in contrast to the HER2-negative (HER−) group (HER− > HER + , p < 0.001). Analysis specific to Triple-Negative Breast Cancer (TNBC) revealed a significant association between elevated TNBC levels and increased MCU transcript levels in both DNA microarray and RNA sequencing data. Across various breast cancer subtypes, MCU expression was consistently lower in normal tissue compared to other subtypes. In summary, these findings collectively underscore the prognostic significance of clinicopathological parameters in breast cancer.

**Fig. 3:**
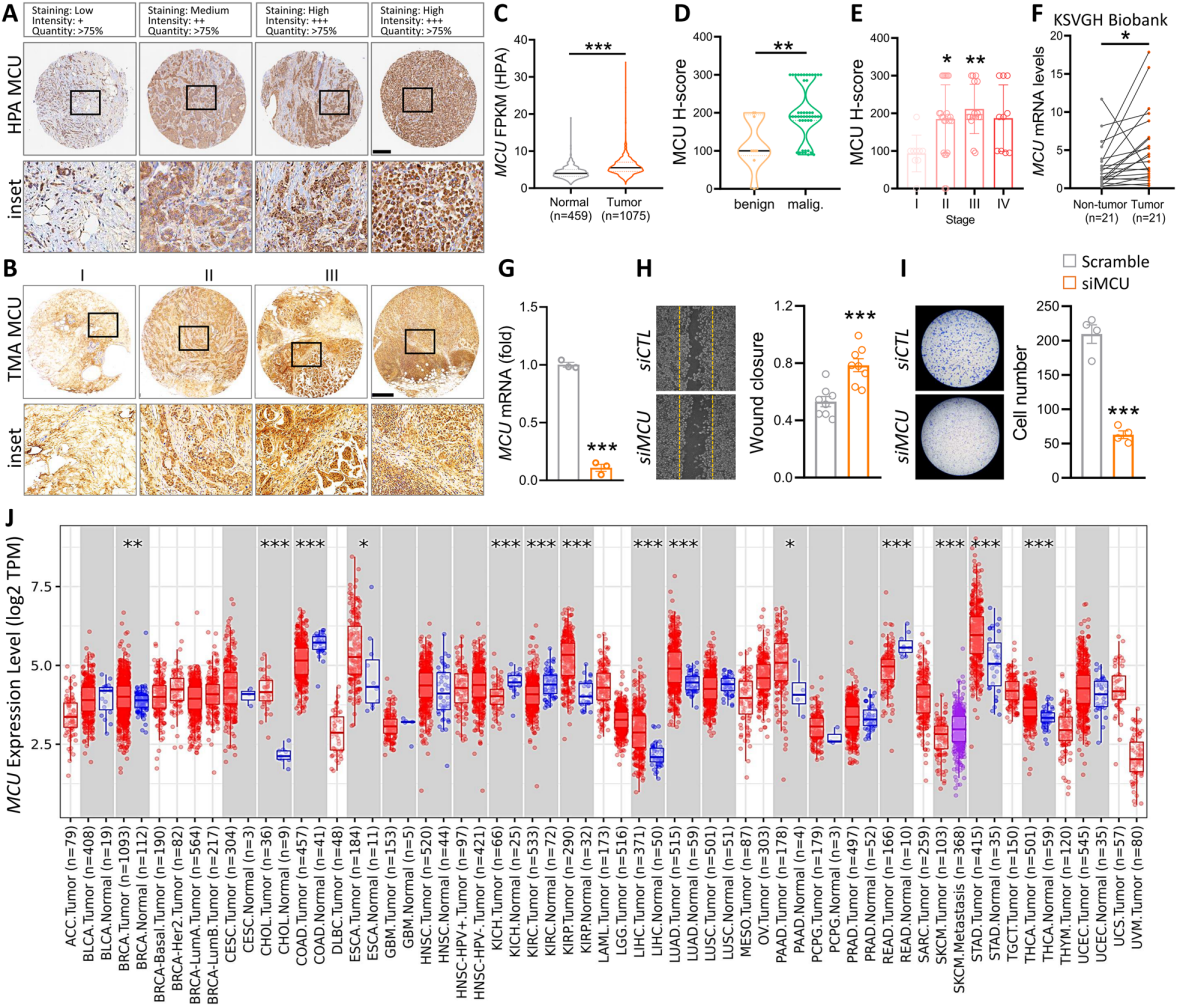
Examination of the pathological alterations of MCU in clinical breast cancer. **A** Immunohistochemical assessment of MCU protein expression levels in BRCA tissue samples from diverse patients based on the Human Protein Atlas. **B** Depicts representative images of MCU expression in breast cancer tissues at distinct staining stages. **C** MCU expression levels in breast cancer were evaluated in tumor and non-tumor tissues using data from the Human Protein Atlas. **C** MCU expression levels in breast cancer were depicted in benign and malignant violin plots. **E** Comparative analysis of MCU expression in BRCA, with box plots illustrating expression levels across different stages of the disease. **F** qPCR analysis of MCU in 21 paired BRCA and non-tumor tissues, denoted as N and T for non-tumor and tumor tissues, respectively. **G** Quantitative PCR demonstrates a significant decrease in MCU expression in breast cancer cells transfected with siMCU. **H** Assessment of wound healing in the MDA-MB-231 cell line through a wound healing assay. **I** Evaluation of breast cancer cell invasion using the MDA-MB-231 cell line in a Transwell assay. (J) MCU expression levels in different cancer types, with comparisons between tumor and normal tissues, highlighting statistical significance using asterisks. *p < 0.05, **p < 0.01, ***p < 0.001. Scale bar = 500 µm

To confirm the accuracy of the multicomponent analysis, we then explored the protein levels of MCU in breast cancer through the Human Protein Atlas (HPA) database, which showed higher levels in late stages than in early stages (Fig. 3A). Further validation was performed using 59 samples obtained from BRCA patients for Tissue Microarray (TMA) analysis. Immunohistochemistry (IHC) analysis of MCU demonstrated a significant increase in MCU levels as breast cancer advanced and became more malignant with increasing stages (Fig. 3B). The quantitative results from HPA data underscored a substantial elevation in MCU expression (Fig. 3C). Corresponding H-score response results demonstrated abundant MCU expression across benign and malignant tumors, as well as various cancer stages (Fig. 3D, E). Moreover, our investigation included the analysis of 21 breast cancer biopsy samples from the human biobank, affirming that MCU levels were notably higher in tumor tissues compared to non-tumor tissues (Fig. 3F). Subsequently, to illustrate MCU's involvement in BRCA progression, we conducted migration and invasion assays post-MCU knockdown in the MDA-MB-231 cell line (Fig. 3G). The findings revealed that MCU suppression impeded MDA-MB-231 breast cancer cell migration, as confirmed by the wound healing assay (Fig. 3H). Furthermore, MCU deficiency decelerated the invasion of breast cancer cells (Fig. 3I). These findings collectively suggest that MCU functions as a tumor promoter by facilitating BRCA cell migration and invasion. In order to delve deeper into the potential clinical significance of MCU, we examined its expression across various cancer types and assessed its prognostic relevance. Similar expression patterns of MCU were observed in multiple cancer types, including Breast Invasive Carcinoma (BRCA), Cholangiocarcinoma (CHOL), Esophageal Carcinoma (ESCA), Uterine Corpus Endometrial Carcinoma (UCEC), Kidney Renal Papillary Cell Carcinoma (KIRP), Liver Hepatocellular Carcinoma (LIHC), Lung Adenocarcinoma (LUAD), Pancreatic Adenocarcinoma (PAAD), Stomach Adenocarcinoma (STAD), Thyroid Carcinoma (THCA), and similar BRCA-like cancers (Fig. 3J).

### Examining the MCU transcriptome through single-cell RNA sequencing libraries

**Fig. 4:**
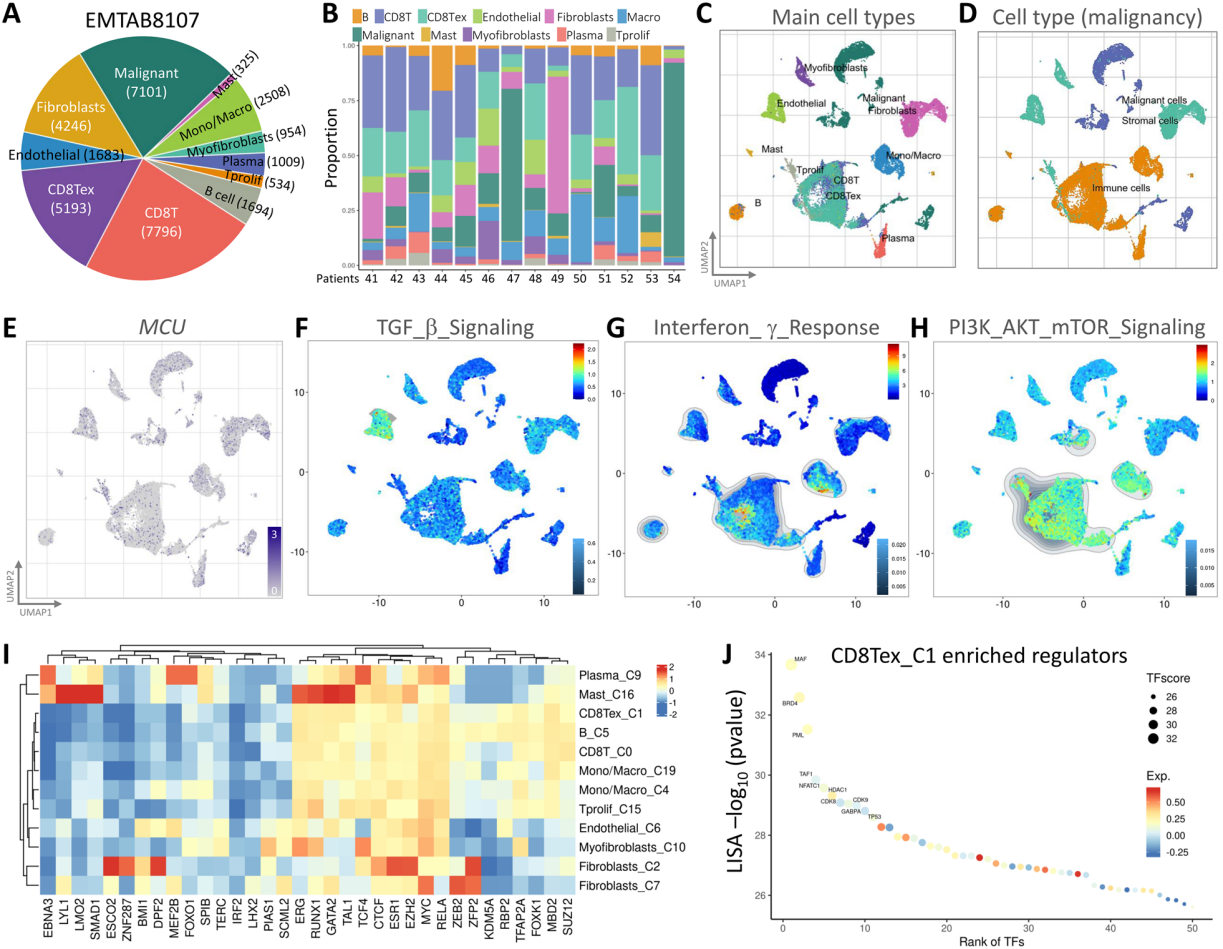
Utilizing single-cell RNA sequencing analysis for the identification of immune cell populations. **A**–**B** Illustrate the relative proportions of each cell type in the public dataset and the integrated immune cell proportions in the EMTAB8107 dataset. **C**–**D** Employ the unified flow approximation and projection (UMAP) technique to visually represent BRCA cells, color-coded based on main cell types and malignancy. **E** Visualizes the expression clusters of MCU through UMAP plots, subsequently subjected to gene set enrichment analysis (GSEA) for **F** TGFβ, **G** interferon γ, and **H** PI3K/AKT/mTOR signaling. **I** The heatmap depicts the expression of the MCU gene and cell type biomarkers in different single-cell type clusters of tissues. **J** Highlights CD8Tex cells as key regulators of transcription factors in this cell cluster

To scrutinize the transcriptome of MCU at the single-cell level within gastric cancer and investigate the diversity of cell types within the gastric cancer microenvironment, we conducted an analysis utilizing an available single-cell RNA sequencing database from breast cancer (EMTAB8107) (Fig. 4A). This database originates from samples of human breast cancer patients and encompasses 11 distinct cell types. Following the elimination of batch effects and quality control, we employed UMAP plots to identify 11 crucial cell populations (Fig. 4B). Utilizing the highest differentially expressed genes in each cluster, we identified cell type-specific markers, crucial for subsequent cell type classification (Fig. 4C, D). Upon scrutinizing the expression and distribution of MCU in these single-cell RNA-sequencing databases, we observed heightened MCU expression in regions corresponding to the inflammatory response model (Fig. 4E). Furthermore, the activation of TGFβ Signaling, Interferon γ Response, and PI3K/AKT/mTOR Signaling pathways was generally observed (Fig. 4F–H). To explore the potential association between BRCA and MCU, we delved into the gene regulation of cell clusters by various transcription factors and pinpointed those strongly linked to CD8 T cells (Fig. 4I, J). It is important to note that T cells exhibit tissue residency and display either pro- or anti-inflammatory functions.

### MCU upregulation is associated with immune cell infiltration

**Fig. 5:**
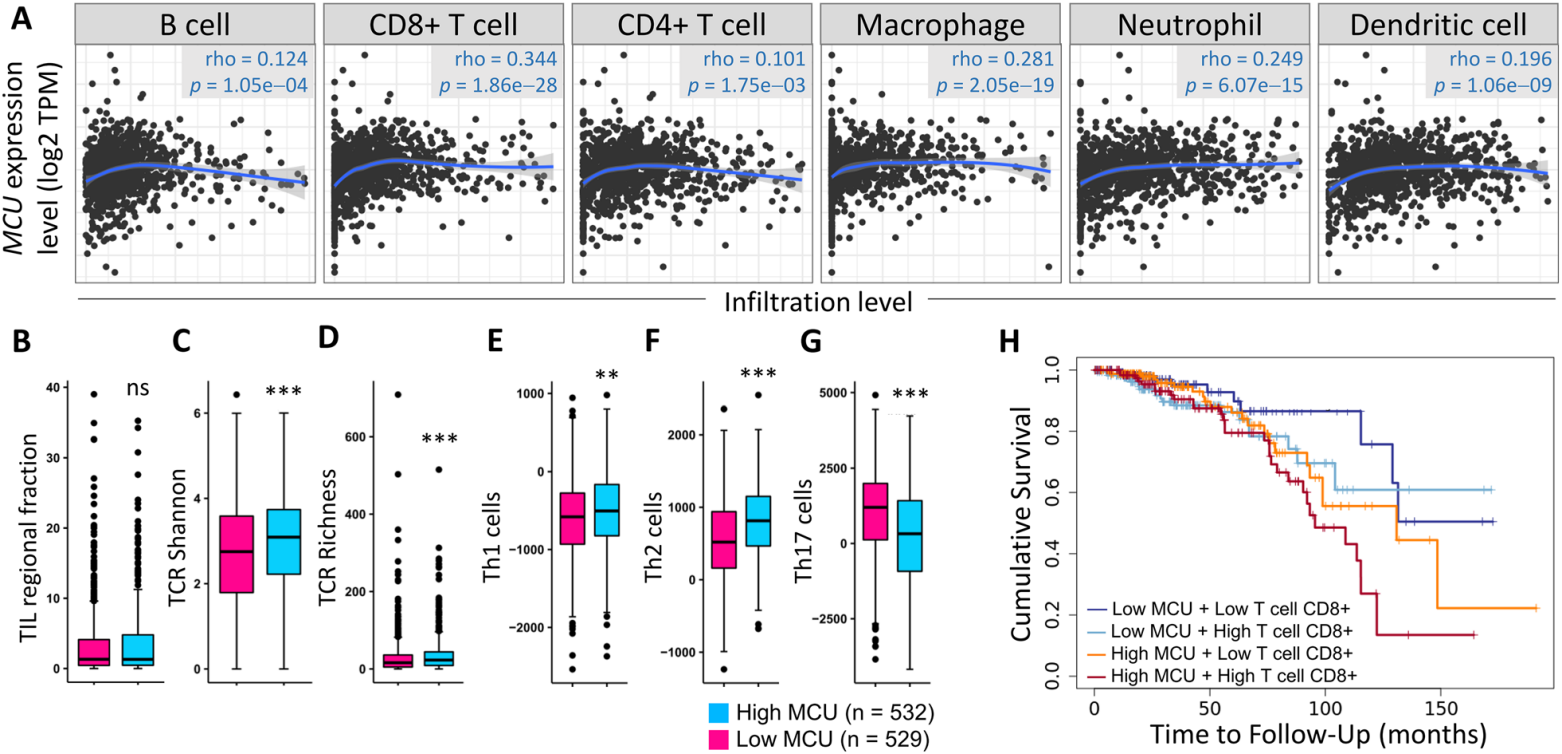
Correlation of MCU expression with immune infiltration level in BRCA. **A** The correlation between MCU expression level and immune infiltration. **B**–**G** Displays changes in transcript levels among different MCU levels and immune cells. **H** Kaplan–Meier plots illustrating the survival differences of macrophages based on different MCU expression levels.*P < 0.05, **P < 0.01, ***P < 0.001

**Fig. 6:**
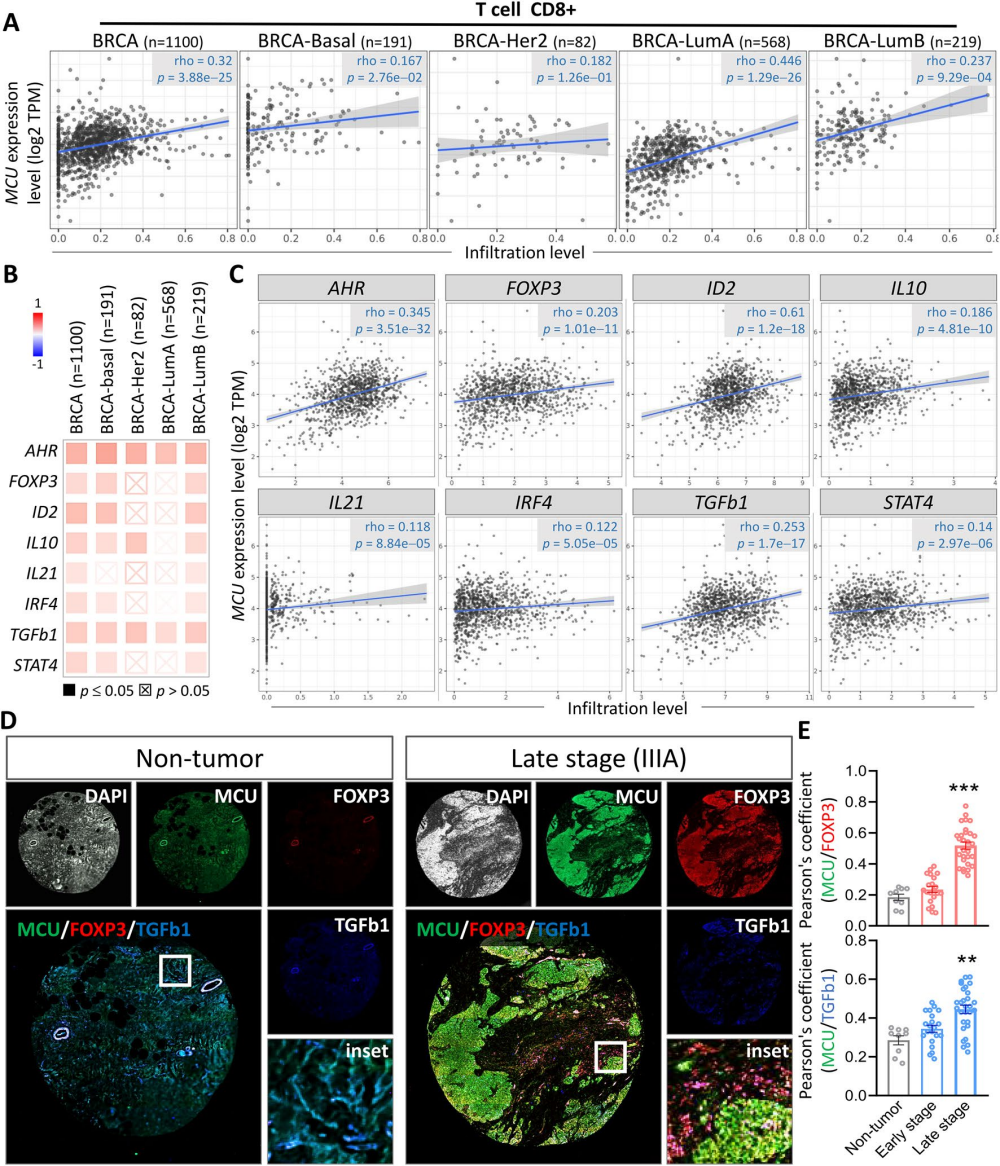
Investigating the association between MCU and immune infiltration in BRCA. **A** Assessing the relationship between MCU levels and T cell CD8 + . **B**–**C** Investigating the correlation between MCU and genes related to T cell CD8 + . **D** Investigating the association between MCU and T cell biomarkers in breast cancer biopsies. Utilizing comprehensive immunofluorescent labeling on tissue microarrays (TMA) with DAPI, MCU, FOXP3, and TGFb1, followed by panoramic tissue scanning. Pearson's correlation coefficient was employed to depict the degree of co-localization between MCU and FOXP3 **E** as well as TGFb1 **F** fluorescent signals. **P < 0.01, ***P < 0.001

MCU overexpression appears to be intricately linked to immune infiltration within the tumor microenvironment of Breast Cancer (BRCA), exhibiting a significant positive correlation with T cell CD8 + (rho = 0.344, p < 0.001; Fig. 5A). In-depth comparative analyses of immune-related functions between the MCU low expression group and the MCU high expression group were conducted. The outcomes indicated no statistically significant differences in TIL regulatory fraction (Fig. 5B). Additionally, a positive correlation was observed between MCU expression and TCR Shannon, TCR richness, Th1 cells, and Th2 cells, while a negative correlation was noted with Th17 cells (Fig. 5C–G). Notably, patients with both high MCU performance and T cell CD8 + infiltration exhibited shorter survival times compared to those with high gene expression alone (Fig. 5H). Further exploration of the correlation between MCU and T cell CD8 + in various breast cancer subtypes also revealed a positive association (Fig. 6A). The investigation extended to studying the T cell CD8 + biomarker, revealing a strong positive correlation between MCU and AHR, FOXP3, ID2, IL10, IL21, IRF4, TGFB1, and STAT4 (Fig. 6B, C). To ascertain the correlation between MCU and T cells in BRCA, we conducted quadruple immunofluorescence labeling, encompassing DAPI, MCU, FOXP3, and TGFb1, across entire BRCA sections in TMA samples. These samples were categorized into non-tumor, early-stage, and late-stage groups, and their fluorescence intensities were assessed using panoramic tissue scanning. As depicted in Fig. 6D, the fluorescence intensities of MCU, FOXP3, and TGFb1 were notably lower in the non-tumor group. Conversely, the late-stage group exhibited a heightened fluorescence overlap ratio of MCU/FOXP3. Similarly, the fluorescence overlap ratio of MCU/TGFb1 was elevated in the late-stage group compared to both the non-tumor and early-stage groups (Fig. 6E).

### The clinical and pathological significance of MCU and its prognostic relevance in patients

**Fig. 7:**
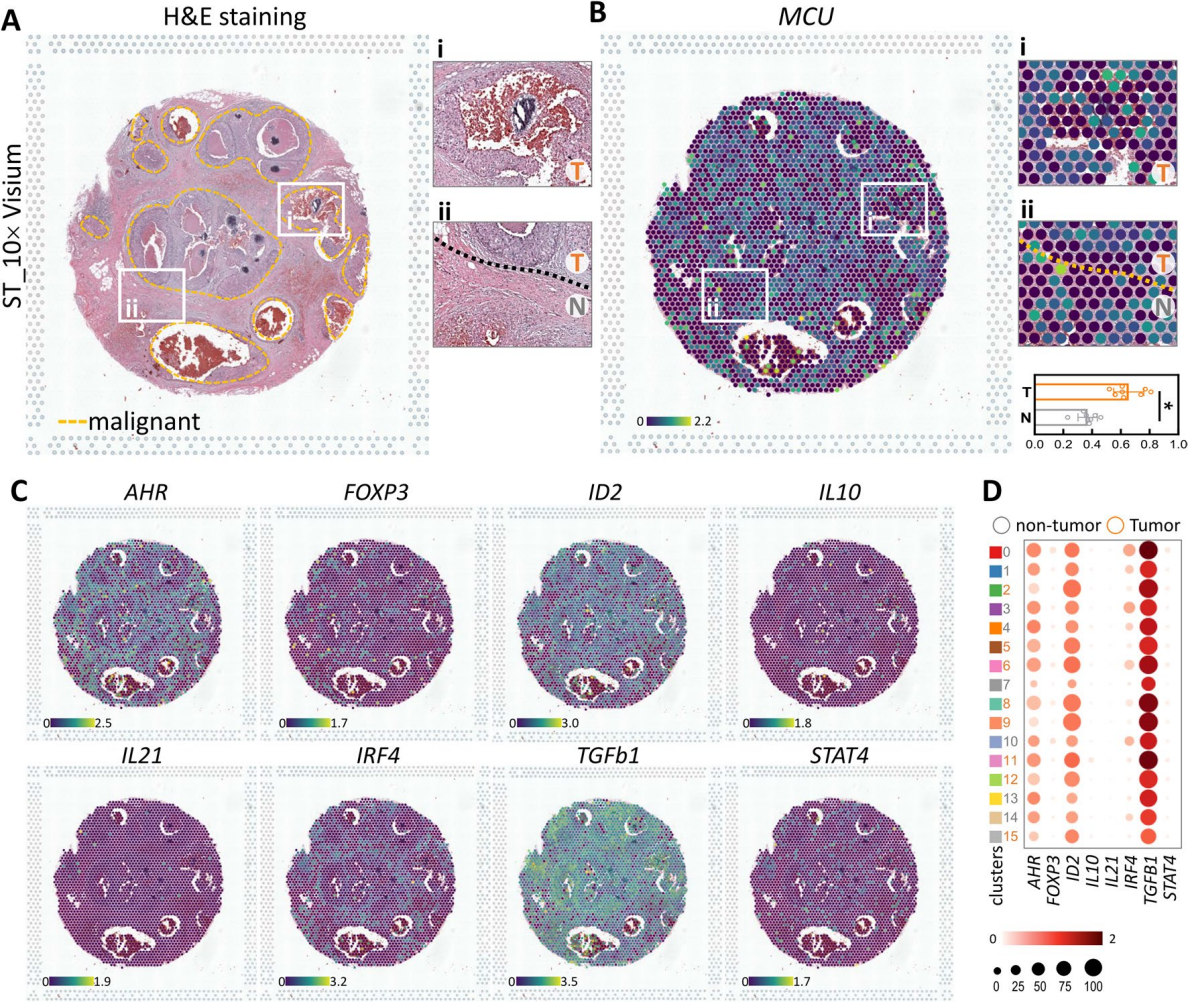
Gene expression in BRCA defined by spatial transcriptomics. **A** Utilizing spatial transcriptomics, tissue sections were analyzed to identify clusters, accurately aligning them with morphological features observed in hematoxylin and eosin staining and cluster mapping. Malignant areas are outlined by yellow dotted lines, and magnified images of gene variants in boxes i and ii are presented. **B**–**C** Spatial distribution and genetic changes involving MCU, AHR, FOXP3, ID2, IL10, IL21, IRF4, TGFb1, and STAT4 in different tissue sections were visualized using 10 × Visium Spatial Gene Expression. Bars indicate tumor versus non-tumor transcript levels. **D** Dot plots illustrate gene expression levels across various clusters. *P < 0.05

To map transcriptomic signatures onto H&E-stained histological sections of human breast cancer tumors (PMID: STDS0000049) (Fig. 7A), we employed spatial transcriptomics techniques. This innovative approach utilizes the sequencing of spatially localized barcodes to directly correlate transcriptomic signatures with histological images. Our analysis encompassed a total of 2518 counts, each possessing its unique expression signature, superimposed on local barcode-based tumor histology images. Employing an unsupervised clustering approach, we categorized points based on their gene expression, with each cluster denoting a specific cell type identified through known marker genes and underlying histology. Comparing Mitochondrial Calcium Uniporter (MCU) with other genes associated with T cell regulation, MCU exhibited higher expression levels in tumor areas compared to non-tumor areas (Fig. 7B). The analysis further revealed that MCU, alongside genes commonly mutated in breast cancer, demonstrated elevated expression levels comparable to key T cell regulators such as AHR, ID2, and TBFb1 (Fig. 7B). MCU expression was notably concentrated in the tumor areas across all 15 clusters, exhibiting highly significant differences in expression, second only to TBFb1. Additionally, the expression levels of these three genes in the high-magnification area were examined to emphasize histological features (Fig. 7C). The Space Ranger algorithm generated 15 unsupervised clusters aligning with known marker genes for each tumor microenvironment cell, superimposed on histological features. Dot plots illustrate the normalized, log-transformed, and variance-scaled expression of various cell clusters (y-axis) and signature genes (x-axis) in Breast Cancer (BRCA) single-cell RNA sequencing data (Fig. 7D).

### CellChat analysis unveiled a continuum of crucial signals associated with cell lineage

**Fig. 8:**
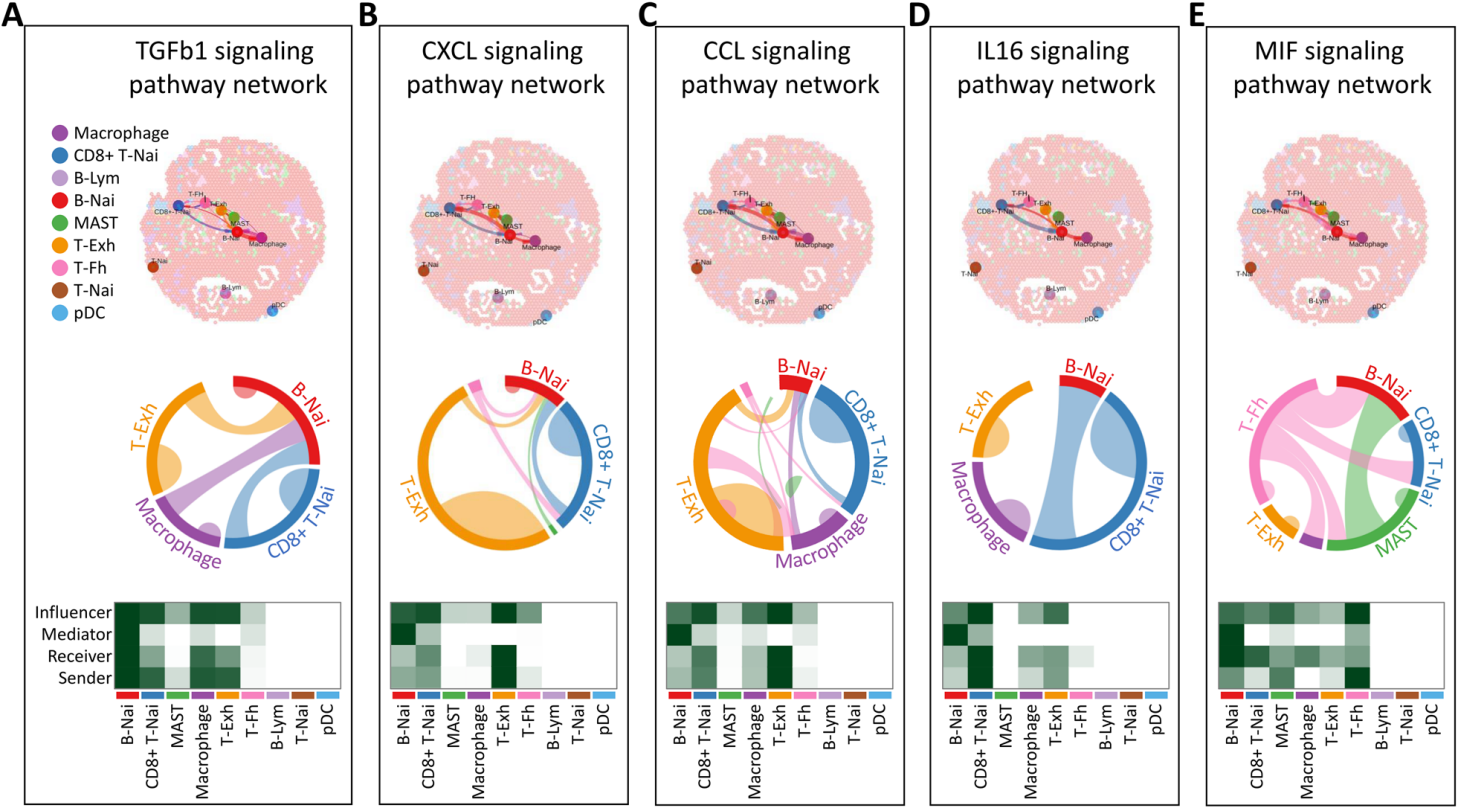
Signaling pathway network associated with T cell CD8 + in the spatial transcriptome of BRCA. **A**–**E** Showcase interactions of the TGFβ, CXCL, CCL, IL16, and MIF signaling pathways with different ovarian cell types. Cell–cell communication is depicted, illustrating interactions between cells, where line thickness reflects the strength of these connections. Chord diagrams for six signaling pathways reveal intricate interactions between cell populations. Heatmaps display network centrality scores for the signaling pathways

To delve deeper, we meticulously identified key senders, receivers, mediators, and influencers within the five signaling networks in cells. This was achieved by computing various network centrality scores for each cell group (Fig. 8A–E). Our focus was on the top five signaling pathways (TGFb1, CXCL, CCL, IL16, MIF) that play pivotal roles in intercellular signaling between different cell populations. It is noteworthy that the CD8 + T-Nai cell cluster emerged as the most significant regulator among these five signal types, with the T-Exh tumor cluster standing out as the primary influencer and recipient of these signals. Specifically, within the TGFb1 signaling pathway, the primary senders and influencers were identified as the B-Nai, CD8 + T-Nai, Macrophage, and T-Exh clusters. Simultaneously, the critical signal receivers were recognized as the B-Nai and Macrophage clusters.

### Pharmacogenomic prediction of effective drugs targeting MCU

**Fig. 9:**
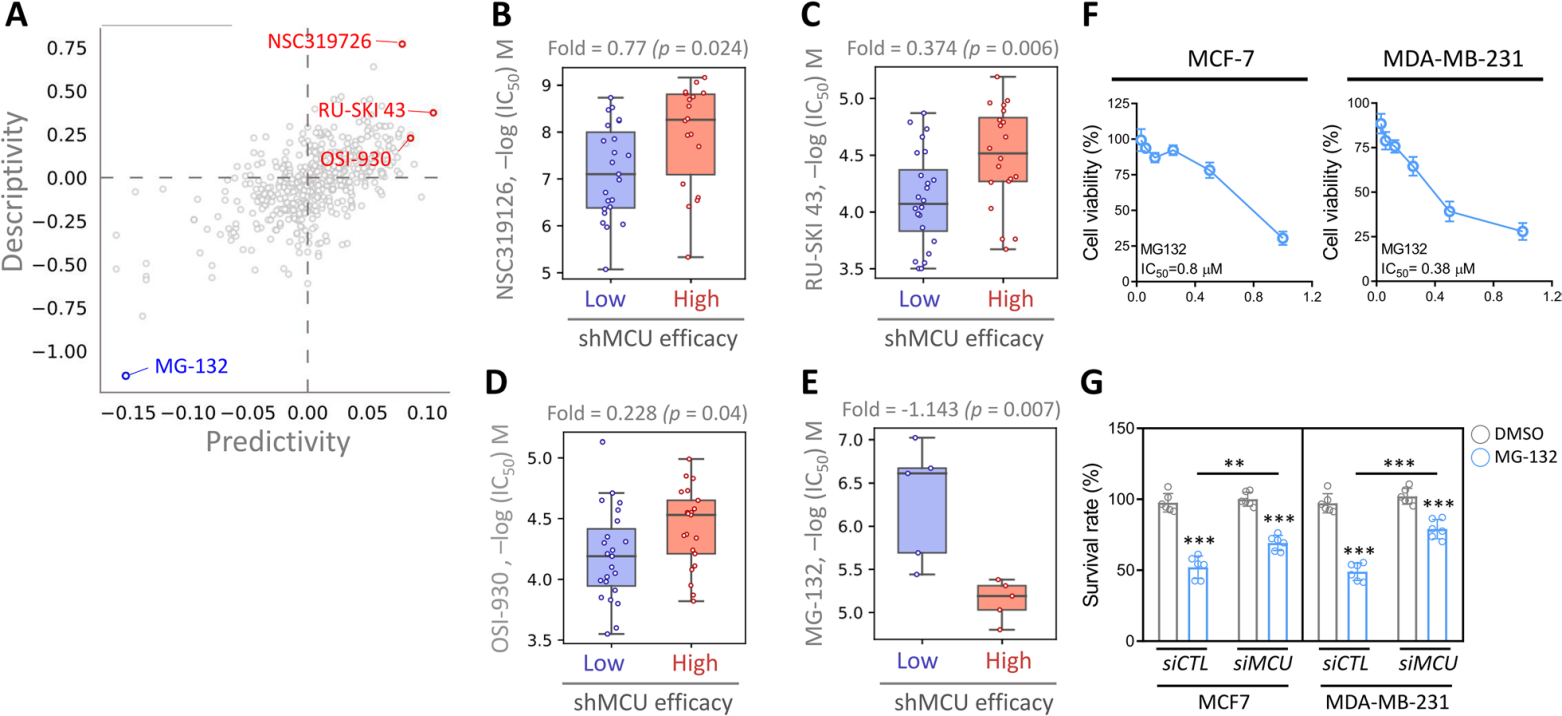
Evaluation of drug sensitivity and cytotoxicity in breast cancer cells. **A** The scatter plot depicts cross-association scores of predictivity and descriptivity, employed to identify potent drugs with efficacy against BRCA cells. To unveil gene signatures and potential drugs, we queried the pharmacogenetics database for the MCU gene. Subsequently, we examined the drug sensitivity of the shMCU gene to various chemical drugs in BRCA cell lines. The boxplots **B**–**E** illustrate the logarithm of the half maximal inhibitory concentration (IC50) values for four drugs, namely NSC319126, RU-SKI 43, OSI-930, and MG-132, displaying altered potency. **F** Evaluation of drug responsiveness across various breast cancer cell lines. **G** Analysis of drug sensitivity following inactivation of the MCU gene. **P < 0.01, ***P < 0.001

To identify potential therapeutic agents targeting Breast Cancer (BRCA), we conducted a thorough analysis of drug responses and the effects of Mitochondrial Calcium Uniporter (MCU) knockdown using shRNA in BRCA cells. A total of 489 drugs were scrutinized, revealing four compounds that displayed significant alterations in potency (Fig. 9A). Notably, BRCA cell lines with low shMCU efficiency exhibited heightened responsiveness to NSC319126 (Fig. 9B), RU-SKI 43 (Fig. 9C), OSI-930 (Fig. 9D), and MG-132 (Fig. 9E). These findings collectively suggest that these drugs hold promise as potential anticancer agents targeting MCU to modulate the growth of BRCA. Further investigations are warranted to explore the therapeutic potential of these drugs and their specific mechanisms of action in the context of BRCA. In the final phase of our investigation, we conducted an intricate analysis to evaluate the response of diverse breast cancer cell lines to the administered drug regimens, meticulously determining the IC_50_ values post-administration (Fig. 9F). This comprehensive assessment revealed notable inhibitory effects on cell proliferation elicited by both drugs. Furthermore, upon specifically targeting the MCU gene in MCF7 and MDA-MB-231 cells, we observed a striking increase in cancer cell viability subsequent to treatment with the same drug dosage (Fig. 9G). These findings underscore the intricate interplay between drug sensitivity and MCU gene expression in breast cancer cells, shedding light on potential mechanisms underlying treatment response variations among different breast cancer subtypes (Additional file 1).

## Discussion

The mitochondrial calcium uniporter (MCU) facilitates the entry of cytosolic calcium into the mitochondrial matrix, playing a pivotal role in regulating ATP production and metabolic fuel selection. Aberrant MCU activity, whether too high or too low, can impact cellular processes, including triggering cell death. While research suggests that mitochondrial Ca2 + uptake plays a pivotal role in the pathophysiology of cancer, the prognostic implications of MCU complex members in malignancies remain inconsistent. Existing literature indicates that MCU-dependent mitochondrial Ca^2+^ signaling may intricately regulate immune cell function [17]. Tregs, relying predominantly on oxidative phosphorylation with moderate glycolytic activity, are particularly reliant on well-regulated mitochondrial function. Tregs can exert inhibitory effects on Tcons by direct contact, impeding Ca^2+^-mediated T cell activation, or indirectly through the release of inhibitory cytokines, such as tumor growth factor-β (TGF-β), interleukin-10 (IL-10), and IL-35. Another avenue involves inhibiting dendritic cell-mediated T cell activation [33]. In COAD, high MCUb expression is associated with increased M1 macrophages and CD8 + T cells, while the proportion of M0 macrophages decreases, illustrating the complex immune landscape in MCU-related contexts [34]. Moreover, bone marrow deletion in MCU mice results in significantly reduced immune cell recruitment during alum-induced peritonitis [35]. Previous studies identified multiple roles for MCU in regulating lymphocyte activation, demonstrating that MCU knockdown enhances B cell proliferation in response to B cell receptor stimulation [36]. In the context of BRCA, functional enrichment analysis revealed that MCU complex-mediated mitochondrial calcium homeostasis is closely linked to T cell function, particularly T cell differentiation and its positive regulation. This underscores the intricate interplay between MCU and immune responses in the context of cancer.

While this article delves into the mutation of MCU in breast cancer, it's crucial to note that MCUb forms hetero-oligomers with MCU, and expressing MCUB alone in the lipid bilayer hardly facilitates Ca2 + exchange across the artificial membrane[37]. MCUb is known to exert a dominant negative impact on uniporter function in various diseases [38]. Hence, we endeavored to scrutinize the genetic variation landscape of MCUb in breast cancer. Our findings revealed that while high and low levels of MCU regulate variations in TP53 and IDH1, only DMD displayed differences between high and low levels of MCUb among the 25 genes linked to breast cancer. TCGA data corroborated that the expression of MCUb in the tumor group exceeded that in the normal group, akin to MCU. However, there was no discernible disparity in overall survival rate. Further subdivision of MCU and MCUb into different subtypes for analysis revealed elevated expression levels of both genes in the tumor group compared to the normal group, mirroring data on cancer stages, major subclass, and metastasis status (Additional file 1: supplementary fig. 1). Despite higher MCUb expression in advanced breast cancer, it does not correlate with mutations in key genes (TP53 and IDH1). Consequently, we speculate that the mutation of MCUb in breast cancer may also involve another assembly protein, EMRE (Essential MCU Regulator), which is indispensable for uniporter function and mediates the interaction between MCU and MICU1 [39]. Our preliminary analysis suggests that the decreased significance of EMRE in the breast cancer group might be attributed to the competitive binding of MCUb with MCU, yet this remains unclear at present.

This comprehensive study integrates multi-omics analysis, clinical validation, and cellular experiments to unravel the novel roles of MCU in BC. Elevated MCU expression in BC emerges as an independent diagnostic biomarker associated with advanced clinical status and predictive of adverse outcomes. Moreover, our findings indicate that the prognostic significance of MCU is modulated by the presence of Th1, Th2, mesenchymal stem cells (MSC), macrophages, CD4 + T-cells, CD8 + T-cells, B-cells, and tumor mutational burden (TMB). Single-cell and whole tissue analyses demonstrate that MCU elevation correlates with the duration of immunosuppression. Importantly, the clinicopathological relevance of MCU extends beyond BC and applies to other cancer types, including CHOL, ESCA, UCEC, KIRP, LIHC, LUAD, PAAD, STAD, and THCA.

This study delves into the potential of MCU as a diagnostic and prognostic biomarker for immune infiltration in Breast Cancer (BRCA). Recognizing the pivotal role of the tumor microenvironment and immune cell infiltration in the host's response to cancer cells, our investigation reveals that the MCU cluster network is intricately involved in inflammation- and immune-related pathways. This suggests that MCU might serve as a novel diagnostic indicator for monitoring the tumor microenvironment. To test our hypothesis, we employed a comprehensive approach, analyzing data from MCU, spatial transcription datasets from the GEO database, and single-cell RNA-sequencing datasets. The analysis demonstrates a positive correlation between T cell infiltration and MCU performance in the BRCA-infiltrated cohort, indicating that MCU may not only provide insights into disease prognosis but also reflect immune status. Path enrichment analysis of MCU positive and negative correlation clustering networks supports this observation. Thus, our findings propose that MCU is intricately involved in the immune infiltration of BRCA and could potentially serve as a prognostic biomarker for assessing the immune response in these cancers. Significantly, this study has clinical implications for prognostic assessment and the management of follow-up immunotherapy. However, it is crucial to acknowledge the limitations of this study. While we screened for suitable drugs and explored different cell lines using pharmacogenomics, selecting four potential targets with the capability to inhibit MCU in BRCA cells, further experimental validation is essential to unravel the molecular mechanisms related to MCU in BRCA. In essence, our study enriches the understanding of MCU's role in BRCA, drawing insights from clinical tumor samples and paving the way for the development of innovative immunotherapy strategies.

## Conclusion

In conclusion, this study advances our comprehension of the diverse and intricate aspects of BRCA molecular biology through an in-depth analysis of MCU expression and its prognostic implications. The observed association between elevated MCU expression and poor prognosis in BRCA suggests potential impacts on the tumor microenvironment and T cell infiltration. As a result, MCU emerges not only as a promising diagnostic and prognostic marker but also as a potential target for immune-related therapies in BRCA. Ongoing research is essential to validate our findings and to unravel the immunomodulatory effects and underlying mechanisms of MCU in the context of BRCA. The study underscores the intricate interplay between MCU and the immune response, highlighting specific genes that warrant further investigation to enhance diagnostic accuracy, refine treatment approaches, and improve overall prognosis for individuals with BRCA.

### Supplementary Information

**Additional file 1**: Alterations and landscape analysis of MCU and MCUb genes in breast cancer.

## Data Availability

Not applicable.
