# Supplementary figures and images for: Spatial and single-cell explorations uncover prognostic significance and immunological functions of mitochondrial calcium uniporter in breast cancer
Source: Cancer Cell Int. 2024 Apr 17;24:140. doi: 10.1186/s12935-024-03327-z (PMC11022417; doi:10.1186/s12935-024-03327-z)

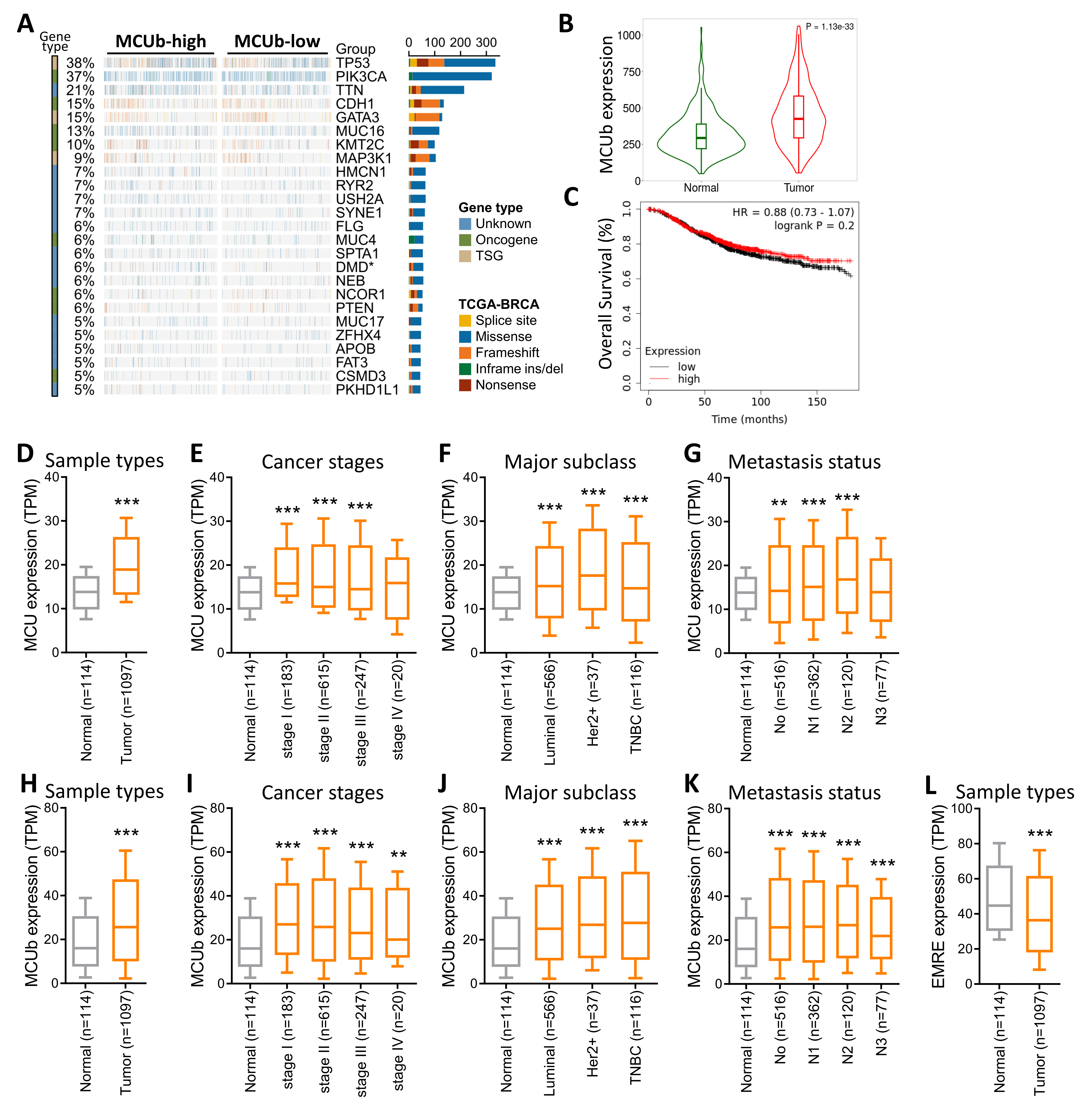

Supplement: Supplementary file 1 — Additional file 1: Alterations and landscape analysis of MCU and MCUb genes in breast cancer. [file 12935_2024_3327_MOESM1_ESM.tif]
